# The role of neuroinflammation in neurodegenerative diseases: current understanding and future therapeutic targets

**DOI:** 10.3389/fnagi.2024.1347987

**Published:** 2024-04-12

**Authors:** Alhamdu Adamu, Shuo Li, Fankai Gao, Guofang Xue

**Affiliations:** Department of Neurology, The Second Affiliated Hospital of Shanxi Medical University, Taiyuan, China

**Keywords:** Alzheimer’s disease, central nervous system, neurodegenerative disease, neuroinflammation, Parkinson’s disease

## Abstract

Neuroinflammation refers to a highly complicated reaction of the central nervous system (CNS) to certain stimuli such as trauma, infection, and neurodegenerative diseases. This is a cellular immune response whereby glial cells are activated, inflammatory mediators are liberated and reactive oxygen and nitrogen species are synthesized. Neuroinflammation is a key process that helps protect the brain from pathogens, but inappropriate, or protracted inflammation yields pathological states such as Parkinson’s disease, Alzheimer’s, Multiple Sclerosis, and other neurodegenerative disorders that showcase various pathways of neurodegeneration distributed in various parts of the CNS. This review reveals the major neuroinflammatory signaling pathways associated with neurodegeneration. Additionally, it explores promising therapeutic avenues, such as stem cell therapy, genetic intervention, and nanoparticles, aiming to regulate neuroinflammation and potentially impede or decelerate the advancement of these conditions. A comprehensive understanding of the intricate connection between neuroinflammation and these diseases is pivotal for the development of future treatment strategies that can alleviate the burden imposed by these devastating disorders.

## 1 Introduction

Neurodegenerative disorder constitutes a substantial and growing global healthcare challenge, which entails the deterioration of neurons in a stepwise manner, resulting in dementia, motor, and other functional impairments (Lamptey et al., 2022). Such adverse conditions like Alzheimer’s disease (AD), Amyotrophic lateral sclerosis (ALS), Parkinson’s disease (PD), and Huntington’s disease (HD), as well as others, are quite detrimental to people and society. However, they have different ways of development and manifestation. Some lead to memory and cognitive impairment whereas others affect movements, speaking, and breathing. Some of these diseases have familial cases linked to causative genes, but many cases are sporadic and etiologically unknown (Pasko et al., 2022). The initial inflammatory response protects and repairs the affected brain tissue in many neurodegenerative diseases (NDs). However, if the insult persists or the inflammation doesn’t subside, it can lead to a chronic inflammatory state, which is a major aspect in the progression of these conditions (Kwon and Koh, 2020).

CNS is a unique system with a regulated immune response compared to the peripheral immune system. Research indicates that a robust inflammatory response in the peripheral system, triggered by factors such as systemic exposure to lipopolysaccharides or viral infections, can result in the infiltration of immune cells from the periphery to the CNS (Sharma et al., 2021). From there, this infiltration prompts neuroinflammation and nerve cell destruction. Initially, the immune response starts with microglia activation, whereby pro-inflammatory messengers are released by microglia into the Blood-brain barrier (BBB), which weakens. Therefore, T-cells and macrophages from the peripheral immune system migrate into the CNS (Ronaldson and Davis, 2020). Importantly, these peripheral immune cells share functional characteristics with microglia, including the expression of toll-like receptors (TLRs), which enables their activation by aggregated proteins or pathogen-related molecular patterns. Therefore, the enhanced permeability of the BBB increases the likelihood of peripheral macrophage involvement in CNS inflammation. Acute neuroinflammatory reaction benefits CNS since it triggers an innate immune response that limits damage. Chronic inflammation is evidenced by continuous microglial activation that leads to the production of inflammatory molecules. This increases oxidative and nitrosative stress levels (Sochocka et al., 2017). It sustains the inflammation cycle, thereby extending the duration of inflammation, which is detrimental to NDs. Neuroinflammation and microglia activation are central events leading to neurodegeneration. For example, in AD, the activated microglia are characterized by increased levels of IL-1 and exist near amyloid beta plaque and neurofibrillary tangles (Zhang et al., 2021). On the contrary, in patients with ALS, they are found in the areas of motor neuron degeneration. The abnormal phosphorylation of the microglial activated by IL-1 results in the increase of tau proteins (Li et al., 2003). This review seeks to examine the work of neuroinflammation in NDs. Experimental studies have provided evidence for neuroinflammation as a core pathophysiological process in many CNS diseases over the last few decades. Prevention of neuroinflammation is an important goal in treating these conditions. However, the exact pathways of neuroinflammation have not yet been fully clarified.

## 2 Inflammation’s role in neurological disease

Inflammation is a normal part of the body’s defense system against injury or infection. Inflammation within the nervous system or neuroinflammation is a complex phenomenon with both protective and detrimental effects in various neurological diseases. Initially serving as a natural defense mechanism during acute events such as infections, injuries, or trauma, inflammation helps contain the damage, clear debris, and initiate the healing process (Chen et al., 2018). However, in chronic conditions, sustained neuroinflammation can be detrimental, perpetuating a cycle of neuronal damage and degeneration. Chronic neuro-inflammation is characterized by excessive activation of glial cells, including microglia and astrocytes. These cells release pro-inflammatory cytokines, chemokines, and ROS, which can worsen neuronal damage and lead to progressive neural deterioration seen in diseases such as AD, PD, HD, and MS (Gąssowska-Dobrowolska et al., 2023).

This disruption allows peripheral immune cells to infiltrate the CNS, thereby perpetuating inflammation and neuronal damage. Moreover, factors such as gut microbiome imbalances and the effects of aging add complexity to the interaction between inflammation and neurodegenerative processes (Jain et al., 2023).

Understanding the intricate balance of neuroinflammation is essential for developing targeted treatments aimed at modulating the immune response to effectively prevent and treat neurological diseases.

## 3 Mechanism of neuroinflammation

Neuroinflammation denotes the innate and adaptive responses to harmful factors such as infections, ischemia, stress, and trauma (Gorji, 2022). Within the context of neuroinflammation, it is hypothesized that four distinct features function as defining hallmarks: elevated cytokine release, microglial cell activation, migration of peripheral immune cells, and localized tissue damage (Konsman, 2022). The response is triggered by microglia, astrocytes, and immune cells (monocytes, neutrophils, lymphocytes) releasing inflammatory mediators like cytokines, histamines, and ROS (DiSabato et al., 2016). Neuroinflammation initially serves as a defensive reaction, but now studies have proven that prolonged or excessive inflammation is a chief contributor to the development of various neurological disorders, especially degenerative diseases (Kwon and Koh, 2020). Diverse approaches to neuroinflammation triggers are listed below.

### 3.1 Microglial activation

**FIGURE 1:**
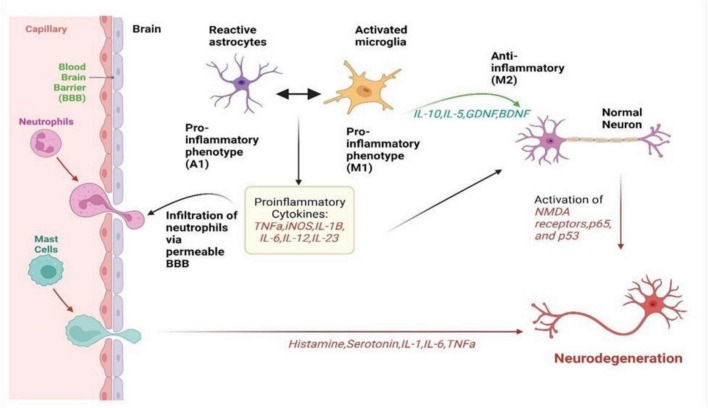
It summarizes how neuroinflammation unfolds via the activation of microglia, astrocytes, and peripheral immune cells in neurological disorders. It visualizes the complex interplay between these cells, illustrating their roles in releasing inflammatory signals, causing a cascade of inflammatory responses, and collectively affecting neuronal health. It highlights the intricate network of interactions driving neuroinflammation, offering insight into the mechanisms underlying disease progression and potential targets for therapeutic intervention (Paul et al., 2021).

Microglia, being a resident immune cell of the CNS, represents up to 20% of the glial population. Microglia are normally activated when the CNS is infected or injured and they move toward the site of infection (Qin et al., 2023). They are also responsible for destroying infected cells and releasing different types of cytotoxins that help in fighting invasive agents. On the other hand, if microglia become hyperactivated, the released substances may prove to be toxic to their neighboring normal tissues (Gao et al., 2023). Microglia can either activate nerve growth or induce neurotoxicity depending on their state of activation. As summarized in Figure 1, in accordance with the classification of macrophages, microglia are commonly grouped into M1 microglia which are classically stimulated and alternatively activated M2 microglia (Guo et al., 2022). TLRs and the gamma interferon signaling pathway induce the production of the inflammatory M1 subtype, characterized by the release of various pro-inflammatory cytokines such as IL-1, IL-6, IL-1β, TNF-α, and NF-kappaB and chemokines (Chen et al., 2023). Additionally, M1 microglia expresses NADPH oxidases and matrix metalloproteinases-12 (Yang H. et al., 2020). In contrast, the neuroprotective M2 subtype promotes the expression of Arg-1, secretion of growth factors, and the release of anti-inflammatory cytokines such as IL-10 and TGF-β (Radandish et al., 2021). This distinction between M1 and M2 microglia underscores the intricate regulatory roles these cells play in neuroinflammation and neuroprotection within the CNS.

Glucagon-like peptide 1 Receptor (GLP-1R) is emerging as an anti-inflammatory agent. Activated GLP-1 reduces microglia-induced neuroinflammation by shifting from M1 to M2 subtypes, both *in vitro* and *in vivo*, and inhibits the generation of reactive astrocytes (Diz-Chaves et al., 2022). Under physiological conditions, microglia engage in a diverse range of activities that enable them to detect and evaluate inflammatory signals in their environment, promote synaptic plasticity and neuronal function, and remove cellular debris through phagocytosis (Shao et al., 2022). These activities are crucial for maintaining overall brain health and function. Microglia can become hyperactive in pathological states and assume significant roles in neuroinflammation, and the development of CNS disorders including acute injuries such as stroke and head trauma as well as AD, PD, and chronic traumatic encephalopathy (Dhaiban et al., 2021). Microglial activation emphasizes the versatile and dynamic nature of this response in the CNS during either a chronic or an acute challenge. To minimize neuroinflammation, it is essential to inhibit this abnormal microglial activation, which could serve as a new treatment approach for various CNS disorders.

### 3.2 Astrocytes

Astrocytes are star-like cells, a subtype of glial cells situated in the brain. In the initial stages of research into the heterogeneity of astrocyte responses, two distinct types of reactive astrocytes were identified: inflammatory/neurotoxic astrocytes, referred to as “A1” astrocytes, and neuroprotective astrocytes, referred to as “A2” astrocytes (Reid and Kuipers, 2021). The JAK/STAT3 pathway, NF-κB pathway, MAPK pathway and calcineurin pathway are all involved in initiating and regulating astrocyte activity (Giovannoni and Quintana, 2020). While NF-κB, Calcineurin pathway, and MAPK pathway play a significant role in controlling reactive astrocytes, they appear to modulate rather than initiate this process. They play multifaceted roles in neuroinflammation through numerous processes that affect the pathophysiology of NDs (Ben Haim et al., 2015). Astrocytes are integral to maintaining homeostasis of the CNS and respond dynamically to injury or disease. During neuroinflammation, astrocytes become reactive, a state characterized by morphological changes and upregulation of glial fibrillary acidic protein (Hol and Pekny, 2015). In their reactive state, astrocytes can produce and release a range of inflammatory mediators, including cytokines and chemokines, that influence the behavior of the surrounding neurons and immune cells. This can be protective, promoting the clearance of pathogens and debris and facilitating repair processes (Ding et al., 2021).

Astrocytes have been found to increase the expression of transmembrane receptors for IL-17 and TrkB in response to neuroinflammation. Upon binding of IL-17 to its receptors, the recruitment of NFκB activator 1 may occur, leading to the production of pro-inflammatory cytokines (Kwon and Koh, 2020). However, persistent, or dysregulated astrocyte activation can also contribute to chronic inflammation. Neurodegenerative processes are often characterized by the ongoing astrocytic release of proinflammatory factors that exacerbate neuronal damage. Moreover, astrocytes regulate the blood-brain barrier, and during neuroinflammation, their processes can alter BBB permeability, potentially permitting unwanted immune cells and neurotoxic substances to enter the brain parenchyma (Zang et al., 2022). Astrocytes are crucial in the process of myelination by releasing substances that encourage the development of oligodendrocytes and the formation of myelin. Moreover, these cells ensure the provision of energy and essential nutrients to the oligodendrocytes by maintaining metabolic regulation. Additionally, astrocytes protect both oligodendrocytes and myelin by maintaining the integrity of the BBB (Hu et al., 2023).

Astrocytes also affect neurodegeneration through their roles in CNS metabolism and neurotransmitter homeostasis. In neurodegenerative conditions, astrocytic dysfunction can disrupt the metabolic support of neurons, aggravate excitotoxicity by limiting glutamate uptake, and alter synaptic function, all of which exacerbate neuronal loss and cognitive decline (Satarker et al., 2022). Additionally, astrocytes interact with other glial cells such as microglia, potentially escalating neuroinflammatory responses and influencing disease progression. Their capability for phagocytosis, which is essential for cleaning up myelin debris and dead cells, can also become maladaptive, leading to sustained inflammation and the inhibition of regenerative processes (Sun et al., 2023).

### 3.3 Infiltration of peripheral immune cells

Peripheral immune cells such as Neutrophils, Monocytes, NK cells, Dendritic cells, T-cells, and even B-cells play crucial roles in the immune response (Kanashiro et al., 2020). In immune response, neutrophils engulf microbes around infected areas via phagocytosis. Neutrophil migration into CNS has been noted under certain pathological conditions, including neurodegeneration (Turner and Sharp, 2016). These studies suggest that these neutrophils facilitate the breakdown of the BBB through the release of free radicals, proteolytic enzymes, and matrix metalloproteinases (Guo et al., 2021). Secondly, post-neutrophil activation and BBB damage lead to the release of neutrophil extracellular traps into the extracellular space, which further impairs the integrity of the BBB (Zang et al., 2022). Such structures are complex webs formed from neutrophil degranulation. Neutrophilic migration to the cortex and hippocampus releases IL-17 that causes cytotoxicity and BBB disruption. This in turn attracts more neutrophils to the site and intensifies the damage (Spiteri et al., 2022). Monocytes are a type of white blood cell found in the CNS and it’s differentiated into monocyte-derived macrophages and monocyte-derived dendritic cells (Ochocka and Kaminska, 2021). Monocyte-derived macrophages enter the CNS through a compromised or intact BBB. These infiltrating blood-borne monocytes have a crucial role in demyelination processes within the CNS and contribute to the secretion of neurotrophic and anti-inflammatory factors that regulate neuropathic events.

Monocyte-derived macrophages contribute toward reduced acute and chronic microglia-mediated inflammation. In pathological conditions, there is migration of NK cells toward the CNS by chemokines (Rivas-Fuentes et al., 2021). The CX3C chemokine ligand 1 produced by neuroinflammation is an essential factor in guiding the CX3CR1 positive NK cell to the brain (Maghazachi, 2013). In neuroinflammation, NK cells engage with glial cells affecting it and affecting the physiology of CNS by getting rid of glial cells and releasing the IFN-γ (Cabeza-Cabrerizo et al., 2021). Dendritic cells express MHCII and CD11c in CNS and this expression is used to identify the site of dendritic cells in the brain (Kennedy and Silver, 2015). The release of chemokines and adhesion molecules enables peripheral dendritic cells to migrate to the meninges or choroid plexus, where they can recognize antigens and present them to T-cells, leading to T-cell activation. It is important to note that the number of B-cells in the CSF increases substantially in CNS inflammation. In addition, B-cells significantly increase their number in the brain parenchyma and perivascular space (Spiteri et al., 2022). This draws attention to the role played by peripheral immunoglobulin cells such as dendritic cells and B–cells in the sophisticated immune reactions occurring in inflamed CNS.

### 3.4 Cytokine production

Cytokines are very important messengers between the immune systems which includes innate and adaptive immunity. Several cells in the CNS can synthesize cytokines such as glial cells, blood components, and peripheral tissues (Seif et al., 2017). The effects of cytokines are mediated through ligands binding to receptors and triggering either JAK-STAT or MAPK signaling pathways. Cytokines can identify such cells and as a result, trigger the migration of further components of immunity and the production of more cytokines (Kempuraj et al., 2017). Cytokine expression is generally low in the peripheral tissue and the CNS. Nevertheless, in response to immunological challenges, peripheral cytokines, and peripheral immune cells, for instance, T cells, cross a protective BBB through various active transport mechanisms. Besides, peripheral cytokines diffuse across the BBB to influence the CNS. In addition, microglia and brain mast cells also secrete cytokines to participate largely in neuroinflammation (Lotfi et al., 2019). Studies have shown that inflammatory cytokines released during neuroinflammation are important in the leukocyte-mediated process. IL-6, IL-23, IL-1β, IFNγ, TNF, and Granulocyte Macrophage Colony Stimulating Factor (GM-CSF) are key cytokines in the neuroinflammation process (Hamilton, 2020). This implies that all cells within the CNS are endogenous IL1β and IL6 producers, contributing toward inflammation. IL-23 is detected through T cells which promotes pathogenic activity. The action of GM-CSF allows inflammatory monocyte-derived cells to cause damage to tissues (Paul et al., 2021).

### 4 Neurodegenerative disease associated with neuroinflammation

Neurodegenerative diseases associated with neuroinflammation encompass a category of diseases that affect the spinal cord and the brain resulting in progressive degeneration of neural tissue and mortality (Sobue et al., 2023). In these diseases, there is a release of immune cells into the CNS causing inflammation and resulting in the degeneration of the neural tissues. Each of these diseases has its own unique set of symptoms and progression, but they all share the common thread of neuroinflammation, which plays a key role in their pathophysiology.

### 4.1 Alzheimer’s disease

**FIGURE 2:**
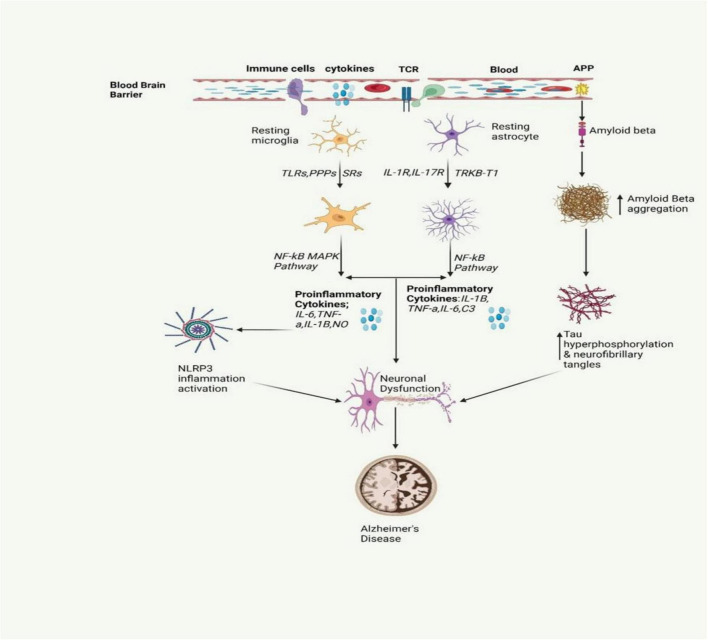
The inflammatory signaling pathway involved in AD is depicted in the figure. It involves the activation of microglia and astrocytes, leading to the release of inflammatory cytokines. This activation also triggers the release of amyloid beta, a protein that is crucial for the formation of neurofibrillary tangles. These tangles and proinflammatory cytokines contribute to neuronal dysfunction, a key characteristic of AD (Gómez-Benito et al., 2020; Pajares et al., 2020).

Alzheimer’s disease stands as the most prevalent cause of dementia, marked by progressive neurodegeneration. The estimated global count of dementia patients is projected to reach 139 million by 2050 (Mohandas et al., 2009). AD is marked by a continuous degenerative process, initially presenting with memory impairment, and subsequently leading to cognitive decline that can affect behavior, language, visuospatial orientation, and ultimately the motor system. As shown in Figure 2, the neuropathological characteristics of AD include extracellular Aβ(amyloid beta) plaques and intracellular hyperphosphorylated tau (p-τ) forming neurofibrillary tangles, accompanied by synaptic and neuronal loss (Sobue et al., 2023). Neuroinflammation in AD occurs when glial cells, mainly microglia and astrocytes, get activated. Such an activation prompts the release of inflammatory factors which are mainly cytokines and chemokines which in turn surround the senile plaques and damaged neurons in the brain (Clayton et al., 2017).

Investigations show clear evidence of microglial inflammation at the brain level among humans with AD, which is been visualized *in vivo* via PET imaging. Pro-inflammatory cytokines are detected at higher levels of the blood serum and postmortem brain tissues among the AD patient and specifically, Aβ can be used to activate the brain’s immune cells. Microglia under activation produce certain products that promote neuronal survival and assist in the removal of waste products by stimulating receptors found on triggering receptors expressed in myeloid cells 2, thereby facilitating the degradation of Aβ oligomers (McQuade and Blurton-Jones, 2019). Beta-amyloid oligomers possess a tendency to agglomerate into plaques, which triggers the activation of microglia. However, if these immune cells fail to effectively remove Aβ, they become chronically activated and intensify the aggregation process. This persistent activation results in the secretion of pro-inflammatory cytokines and neurotoxic substances, which collectively contribute to neuroinflammation and neurotoxicity (McQuade and Blurton-Jones, 2019). Mutations in *TREM2* and *CD33* genes have been discovered to cause a high risk of AD (Griciuc et al., 2019). AD brains experience chronic inflammation due to neuronal loss and involve multiple cell types and molecules (Onyango et al., 2021). These factors combine to make a crucial contribution to the commencement and course of this disease.

### 4.2 Parkinson’s disease

**FIGURE 3:**
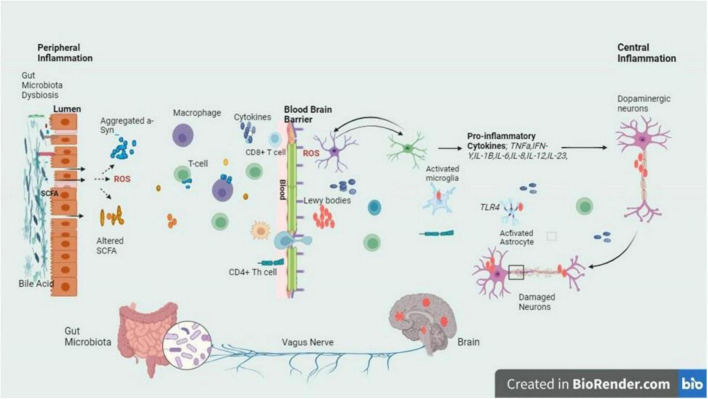
The intricate relationship between the gut and brain axes in PD is delineated by the schematic signaling pathway, with a focus on the inflammatory cascade triggered by dysbiosis at the gut lumen and BBB. Dysbiosis leads to an imbalance in gut microbiota composition, which in turn fosters the release of inflammatory cytokines. These cytokines breach the BBB and infiltrate the brain, where they exert their detrimental effects on dopaminergic neurons. The figure highlights the crucial role of dysbiosis-induced inflammation in contributing to the neurodegenerative characteristic of PD, emphasizing the potential therapeutic targets within the gut-brain axis for mitigating disease progression (Stefanis, 2012; Mey et al., 2023).

Parkinson’s disease follows AD as the most known neurodegenerative condition. It is suffered by more than one in a hundred people who are 65 years old or more. By 2030, the incidence is estimated to double (Gómez-Benito et al., 2020). It is a complex, progressive neurodegenerative disease whereby the number of dopamine-producing cells within the substantia nigra (SN) are irreversibly lost. The deposition of the α – synuclein, a synaptic protein plays an important role in PD, forming aggregates in the form of oligomers or fibrils (Pajares et al., 2020). Aggregates α – synuclein forms clusters and interacts with TLRs, stimulating the activation of microglial cells. Such activation sets off a series of actions, for instance, the synthesis and secretion of pro-inflammatory cytokines. These pro-inflammatory cytokines are overexpressed resulting in the cells’ apoptosis causing the progression of PD (Lull and Block, 2010). However, excessive stimulation of microglia can cause serious, deleterious neurotoxic effects as a result of excessive synthesis of toxic compounds such as superoxide, nitric oxide, and TNF-α (Stefanis, 2012). The overexpression of α-syn or duplication in the *SNCA* gene leads to the buildup of α-synuclein fibrils, major components of Lewy bodies and neurites (Marogianni et al., 2020). In PD, Lewy bodies & α-synuclein-containing neurites cause dopaminergic neuron death, while α-synuclein pathology activates microglia via TLR interaction. This triggers inflammatory responses, pro-inflammatory cytokine release, and NF-κB pathway activation (Figure 3).

Adaptive immunity is also associated with neuroinflammation in PD. The SN of individuals with PD is invaded by both CD 4 + and CD 8 + T cells with CD 8 + T cells being significantly elevated. Cd8 + T-cells may hold the key in early-stage PD and may perhaps come before any detectable pathological α -synuclein in the SN (Ghasemi et al., 2017). In the initial stages of PD, infiltration is observed in the SN, which tends to slow down the progression of the disease. While there have been attempts to measure the number of CD4 + T cells in the blood of PD patients, the results remain controversial. Several studies have found a substantial decrease in CD4 + T cells in PD patients. However, the counting of Treg cells in the blood of PD patients yields inconsistent results, with studies using a non-specific marker of CD4 + CD25 + to identify Treg cells (Ghasemi et al., 2017). Studies have linked gut microbiota imbalance to the development of PD, where dysbiosis increases gut permeability, enabling harmful substances to trigger inflammation. Activation of immune cells by microbial products releases inflammatory molecules that damage dopaminergic neurons through neuroinflammation, facilitated by the vagus nerve (Zhang X. et al., 2023).

### 4.3 Multiple sclerosis, Huntington’s disease and amyotrophic lateral sclerosis

Multiple sclerosis is a chronic immune-mediated disease characterized by demyelination of the CNS (Ghasemi et al., 2017). It affects approximately 2.8 million people worldwide, with a higher incidence in females (Walton et al., 2020). Neuroinflammation in MS is caused by oxidative stress, energy deficits, ion imbalance, mitochondrial damage, and inadequate regeneration processes (Papiri et al., 2023). The disease process includes neuroinflammation, where immune cells infiltrate the CNS, attack myelin sheath, and create demyelinating lesions, which are key in MS pathology and linked to initial neurological symptoms. Axonal transection and chronic demyelination cause permanent damage, ultimately leading to neuron loss and brain atrophy, which signify neurodegeneration (Mey et al., 2023). The disease can manifest in several forms, with relapsing remission being the most common. Although immunomodulatory therapies can temper inflammatory attacks, treatment options for the progressive neurodegenerative phase remain limited, underlining the urgent need for neuroprotective strategies and early interventions to prevent irreversible neurological decline.

**FIGURE 4:**
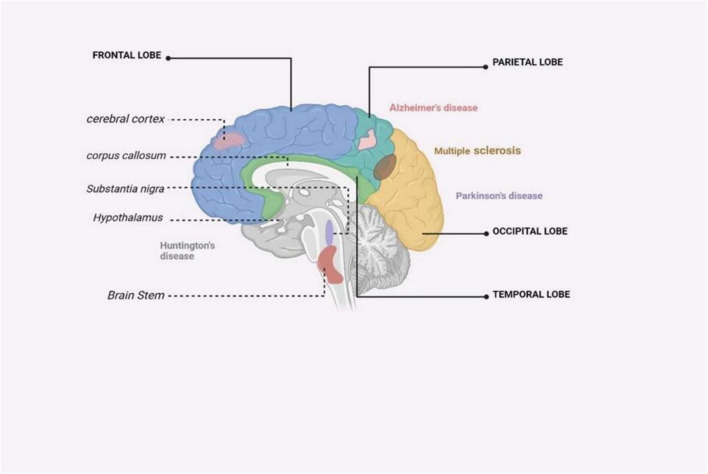
An illustrated summary of the brain regions impacted by major neurodegenerative disorders (Zahid et al., 2019).

HD is a genetic disorder that leads to the progressive degeneration of neurons. It is caused by a repeat mutation in the huntingtin gene (Kim et al., 2021). HD is a neurodegenerative disorder characterized by the selective loss of neurons in the striatum, which results in motor and cognitive impairments and often culminates in death 10–15 years after the onset of symptoms (Jia et al., 2022) (Figure 4). The huntingtin protein in the N-terminal region contains an expansion of glutamine repeats (>36 glutamines), which is believed to be the cause of HD. However, the exact mechanism of neuronal death in HD remains unclear. Several mechanisms, including inflammation, may work together to contribute to the development of HD. Inflammation response alterations are observable even before the appearance of classical HD symptoms. Gaining insight into the impact of inflammation on HD would aid in the development of new treatment strategies for the disease (Jia et al., 2022). Neuroinflammation in HD is stimulated by glial cell activation, resulting in the production of pro-inflammatory molecules. Post-mortem investigations have shown increased levels of pro-inflammatory mediators in various brain regions in individuals with HD (Jia et al., 2022). In HD, neuroinflammation is an early event, evident even before classical symptoms arise, and is a crucial aspect of disease pathogenesis. This inflammatory response includes the activation of microglia and astrocytes, which, under healthy conditions, act in support of neurons (Palpagama et al., 2019). However, in HD, these glial cells, when expressing the mutant huntingtin protein, not only lose their protective abilities but also contribute to neurodegeneration through the release of pro-inflammatory cytokines that damage neurons (Wilton and Stevens, 2020). Although neuroinflammation often has detrimental effects, including exacerbating neuronal death, it can sometimes be beneficial by aiding in the clearance of cell debris (Harry and Kraft, 2008). Comprehending neuroinflammation’s dual function in HD is vital for devising therapies that specifically regulate immune reactions, to alleviate or treat the disease.

ALS is a rapidly progressive neurodegenerative disease characterized by the loss of upper and lower motor neurons. It affects approximately 1.75–3 out of every 100,000 individuals annually (Masrori and Van Damme, 2020). This leads to muscle wasting, weakness, and eventually, paralysis. In the neurodegenerative process of ALS, certain proteins, such as TDP-43, mislocalize and aggregate in cells, contributing to neuronal cell death. ALS is a disease characterized by upper and lower motor neuron loss with a signature feature of cytoplasmic aggregates containing TDP-43, which are detected in nearly all patients (Mejzini et al., 2019). The disease can manifest due to both genetic and sporadic factors, with genetic forms being linked to mutations in specific genes, such as *TARDBP, FUS, SOD1* and *C9orf72* (Mejzini et al., 2019). Inflammation in ALS involves both reactive microglia and astrocytes. Mutant superoxide dismutase 1 released from motor neurons triggers microglial and astrocytic activation, contributing to the inflammatory process (You et al., 2023). Dendritic cells, T cells, microglia, and macrophages produce cytokines and chemokines that promote inflammation. The extent of microglial activation correlates with clinical deficits in ALS (Zhang and An, 2007). Furthermore, neuroinflammation plays a critical role in ALS progression, with activated microglia and astrocytes releasing inflammatory cytokines that can exacerbate motor neuron damage (Mortada et al., 2021). These inflammatory processes not only contribute to the neurodegeneration observed in ALS but may also drive further neurotoxicity. The interaction between neurodegenerative processes and neuroinflammation creates a complex cycle that accelerates ALS progression (Mortada et al., 2021). Despite the growing understanding of these mechanisms, translating this knowledge into effective therapies remains a significant challenge in the battle against this devastating disease.

## 5 Future therapeutic approaches for NDs

Therapeutic approaches for NDs associated with neuroinflammation are diverse and continually evolving. The primary goals of these therapies are to control symptoms, slow disease progression, and promote quality of life for patients. Some common therapeutic approaches include:

### 5.1 Inflammatory targets

Currently, there are no cures for neurodegenerative disorders, and treatments only offer minimal relief for symptoms. However, significant progress has been made in studying the role of neuroinflammation in these conditions, leading to the development of new therapies targeting this critical pathway (Mortada et al., 2021). In other words, a phase II, double-blind, randomized study showed that weekly subcutaneous injections of TNFα(Etanercept) in a tiny number of mild to moderate AD dementia patients had no significant results as compared with placebo (Holbrook et al., 2021).

NLRP3 inflammasome is an important protein complex that activates in response to certain signals such as those involving disturbance of homeostasis-altering molecule patterns, pathogen-related molecular patterns, and danger-associated molecular patterns. Numerous proteins linked to diseases can trigger the NLRP3 inflammasome, including Fibrillar β-amyloid. This protein’s phagocytosis leads to the activation of the NLRP3 inflammasome through the damage of lysosomes and the release of cathepsin B in AD (Holbrook et al., 2021). Activation of the NLRP3 inflammasome by tau monomers and oligomers has been noted to impact tau hyperphosphorylation and aggregation in AD. Additionally, aggregated α-synuclein has been shown to activate the NLRP3 inflammasome in PD. In contrast, mutant SOD1 functions as a damage-associated molecular pattern to activate the NLRP3 inflammasome in ALS (Zhang W. et al., 2023). Suppressing the activation of IL-1β which is controlled by NLRP3 inflammasome, protects transgenic models of AD by effectively stopping the subsequent production of IL-1β, which plays a pivotal role in triggering inflammatory reactions in the brain (Zahid et al., 2019). Inzomelid compounds have been specifically developed to inhibit NLRP3 and have progressed to Phase I clinical trials, showcasing the potential of directly targeting the NLRP3 inflammasome in a clinical setting (Han and Le, 2023). The use of MCC950 (NLRP3 inflammasome Inhibitor) was effective during preclinical conditions where it was tested in the various MS mice models (Sharma et al., 2023). Finally, several animal models of AD, ALS, and HD demonstrate inhibition of pathology and disease progression through the use of C5a inhibitors (Chang et al., 2017). Currently, researchers are investigating the possible anti-inflammatory and immunomodulating functions of such molecules as GM-CSF. The regulatory microglial proliferation is promoted by the GM-CSF-driven effects on dendritic cells. A group of patients treated with 127 amino acids synthetic GM-CSF leukine(Sargramostim) compared to control groups showed an improvement in cognitive function and memory (Chang et al., 2017). Sargramostim has also proven to be safe and tolerable in phase 2 trials for all AD patients. In addition, investigations into the immunomodulatory and neuroprotective effects of Sargramostim are going on. Preclinical and phase I trials revealed a significant improvement in motor disability in PD as well as protection of dopaminergic neurons through Treg induction (Saleh et al., 2022). However, GM-CSF has been controversial in treating ALS with mixed clinical findings showing that GM-CSF might not be important to ALS patients or at least not slow down the disease process (Mortada et al., 2021). Other drugs targeting PPAR-γ and GLP-1 have also been described for their neuroprotective and anti-neuroinflammatory actions besides GM-CSF (Zhang Z. et al., 2023). Modern research suggests that molecules might relieve AD and PD symptoms, by restoring Akt-1 and mTOR, and insulin Pathways in the brain. The use of GLP-1 agonist drugs in a preliminary clinical trial has revealed some improvement in pathophysiology but not sufficient for conclusive evidence (Tong et al., 2016). The ability of PPAR-γ agonists to reduce neuroinflammation, Aβ-42, phosphorylated tau, and synaptophysin, with resulting improvement in spatial memory, as well as motor function, has been demonstrated in AD mouse models (Chang et al., 2017). Two PPAR-γ agonist compounds, T3D-959 and Pioglitazone, have been tested to be safe and tolerable in phase II clinical trials involving patients. Cognition and insulin metabolism have remarkably been improved in AD patients through T3D-959 (Machado et al., 2019). In preclinical studies, it has been shown that pioglitazone effectively reduces neuroinflammation and microglial proliferation in multiple PD models. However, these findings need to be confirmed in clinical trials (Machado et al., 2019).

Anti-inflammatory therapy is a critical aspect of managing NDs, including AD, PD, and ALS. In these diseases, inflammation plays a central and multifaceted role in disease progression (Jung et al., 2019). Consequently, therapeutic efforts have focused on developing drugs that can suppress this inflammatory response. Several approaches have been explored, such as kinase inhibitors, immunotherapy, and repurposing existing medications. Kinase inhibitors target signaling pathways involved in inflammation, while non-kinase anti-inflammatory drugs comprise conventional non-steroidal anti-inflammatory drugs and more targeted therapies that aim to intercept specific aspects of the inflammatory cascade (Zarrin et al., 2021). Therapeutic strategies for these diseases may involve the use of drugs that inhibit the activity of proinflammatory cytokines or the cellular responses they provoke. For instance, NSAIDs can inhibit COX-1 and COX-2, which are involved in the production of pro-inflammatory prostaglandins (Ahmadi et al., 2022). Other targeted therapies may include antibodies or receptor antagonists that specifically neutralize or inhibit cytokines, such as TNF-α, IL-1, and IL-6. These therapies aim to reduce neuroinflammation, which accelerates neurodegenerative pathology (Turner et al., 2014).

However, the development of anti-inflammatory therapies for NDs is complex due to several factors, including the need to cross the BBB, potential side effects, and the precise timing required to intervene without disrupting the natural beneficial roles of inflammation, such as tissue repair and pathogen defense. Therefore, extensive research is needed to optimize these treatments and identify appropriate targets and treatment windows to provide therapeutic benefits.

### 5.2 Toll-like receptors

Toll-like receptors are vital receptors in innate immunity, detecting pathogens or damage-related patterns, and triggering immune responses. Their presence spans between immune and non-immune cells, recognizing specific ligands and activating signaling pathways leading to inflammatory responses (Duan et al., 2022). Their evolutionary conservation underscores their importance in immunity. Dysregulation of TLRs has been implicated in various diseases. TLR2 plays a significant role in NDs by contributing to neuroinflammation and neurodegeneration. In the context of PD, TLR2 has been implicated in the pathogenesis and progression of the condition. An increase in neuronal TLR2 expression correlates with the disease stage, and studies have shown that TLR2 may contribute to the pathology by inhibiting autophagy and promoting the accumulation of α-syn, a protein involved in the formation of Lewy bodies characteristic of PD (Gorecki et al., 2021). Furthermore, in models of PD, TLR2 deficient mice showed reduced accumulation of α-syn and ameliorated motor deficits. Neuronal TLR2 has also been shown to mediate neuron-to-neuron transmission of α-syn, implicating it in the propagation of synucleinopathies (Mazzotta et al., 2023). In ALS, increased levels of TLR2 and TLR4 have been found in the spinal cord of the patient’s postmortem. Its expression, mostly by microglia/macrophages, suggests its role in glia-mediated neuroinflammation and possibly neurodegeneration (Zhang W. et al., 2023). Overall, TLR2 is a key player in the neuroinflammatory response across various NDs, and targeting TLR2 could potentially have therapeutic benefits in these conditions.

TLR4 is a critical component of the immune system and is recognized for its role in mediating the response to pathogen-associated molecular patterns; however, it also has a notable impact on the pathophysiology of NDs (Aboudounya and Heads, 2021). TLR4 is expressed in microglia, the brain’s resident immune cells, and other cell types within the CNS. In neurodegenerative disorders, aberrant activation of TLR4 can contribute to chronic neuroinflammation, a common underlying feature of such diseases. TLR4 has been implicated in the response of amyloid-beta plaques to AD. The binding of Aβ to TLR4 can activate microglia, leading to the release of pro-inflammatory cytokines that contribute to neuronal damage and AD progression (Wu et al., 2022). Similarly, in ALS, the upregulation of TLR4, particularly in glial cells, is associated with enhanced inflammation and neurodegeneration (Fiebich et al., 2018). Experimental models have revealed that genetic or pharmacological inhibition of TLR4 can ameliorate some disease characteristics and extend survival. Although in PD the involvement of TLR4 is less well compared to in AD and ALS, TLR4-mediated neuroinflammation can exacerbate the degeneration of dopaminergic neurons, suggesting that modulation of TLR4 activity might offer therapeutic potential (Conte et al., 2023). Furthermore, in HD, TLR4 activation contributes to an inflammatory environment that facilitates neuronal dysfunction and death.

Overall, TLR4 participates in the cascade of immune responses that result in the production of neurotoxic substances, perpetuating neuronal loss and CNS damage, which are characteristic of NDs (Griffioen et al., 2018). Thus, modulation of TLR4 activity represents a potential therapeutic approach to mitigate neuroinflammation and modify the course of these disorders. However, the precise mechanisms by which TLR4 contributes to the pathogenesis of different NDs and the successful translation of TLR4-targeting strategies into effective treatments require further investigation (Zhou et al., 2020).

### 5.3 Gut microbiota on neurodegenerative disease

The gut microbiota plays a crucial role in the development and progression of NDs through the gut-brain axis, which is a communication system between the gut and the brain (Jain et al., 2023). The gut microbiota is comprised of an extensive array of microorganisms that possess the ability to influence cognitive functioning by transmitting microbial metabolites to the brain via the gut-brain axis or the nervous system. An imbalance in gut microbiota, particularly a surge in detrimental bacteria and a decline in helpful bacteria has been connected to NDs like AD, PD, and HD (Kandpal et al., 2022). It is essential to understand the role of gut microbiota in these diseases to develop novel treatments, including antibiotics, probiotics, and fecal microbiota transplantation, aimed at maintaining healthy gut microbiota and potentially improving outcomes for NDs (Khatoon et al., 2023). Research indicates that changes in the gut microbial composition can result in changes in gut permeability, chronic systemic inflammation, and increased production of amyloid and tau proteins, which are hallmark features of AD, leading to neuroinflammation and neurodegeneration (Kowalski and Mulak, 2019). For example, a decrease in beneficial bacterial populations like Bifidobacteria, known for their anti-inflammatory properties, has been observed in AD patients, alongside an increase in pro-inflammatory bacterial species (Abdelhamid et al., 2022). Several studies have investigated the therapeutic potential of modulating the gut microbiota in AD. Strategies such as the administration of probiotics and prebiotics have been explored to restore a balanced gut microbiota, alleviate inflammation, and potentially enhance cognitive function in AD patients (Ansari et al., 2023).

The increasing interest in the role of gut microbiota in PD is underpinned by mounting evidence suggesting its involvement in the condition’s pathophysiology through the gut-brain axis. Studies have revealed that gut microbiota can impact motor deficits and neuroinflammation, both characteristic symptoms of PD. Specific changes in the gut microbiota’s composition have been linked to the disease and its clinical manifestations (Shen et al., 2021). One hypothesis proposes that misfolded α-synuclein, a protein central to PD pathology, may originate in the gut and migrate to the brain via the vagus nerve. This hypothesis is supported by the presence of gastrointestinal symptoms preceding motor symptoms in many PD patients (Klann et al., 2021). Microbial metabolites, particularly short-chain fatty acids produced by gut bacteria, exhibit neuroprotective effects and impact the immune system, potentially relevant in PD. Animal studies have demonstrated that microbiota transplantation can transfer behavioral phenotypes associated with PD, suggesting a potential role of gut bacteria in modulating neurodegenerative processes (Cenit et al., 2017). Dysbiosis, or imbalance, of the gut microbiota, has been strongly associated with oxidative stress-mediated NDs. Certain strains of probiotics, including Bifidobacterium and Lactobacillus, which are derived from the gut microbiota, possess the capability to produce antioxidants, vitamins, and bioactive molecules. This ability aids in the maintenance of redox homeostasis and helps prevent diseases that are associated with oxidative stress (Wang et al., 2022). Recognizing the role of gut microbiota in these illnesses is important for devising new therapies, including antibiotics, probiotics, and fecal microbiota transplantation to preserve a balanced gut microbiota and potentially enhance outcomes for NDs.

### 5.4 Antioxidants in neurodegenerative disease

Mitochondrial dysfunction and oxidative stress are fundamental functional abnormalities that are closely linked to the pathophysiology of ADs. Recent, placebo-controlled clinical trials conducted within the past few years have explored the potential benefits of antioxidant-based interventions in patients with ADs. Resveratrol, carotenoids, omega-3 fatty acids, vitamin E, and melatonin supplements are examples of substances that have been administered and investigated in these trials (Salehi et al., 2018). There are so many studies that indicate that resveratrol one of the powerful non-flavonoid polyphenols that has been known for its antioxidant, anti-inflammatory, and neuroprotective properties has managed to lower Aβ peptides toxicity and aggregation in the hippocampus, a part of the brain that is involved in AD patients (Li et al., 2020). Resveratrol also increases neurogenesis, protects the BBB, and stops hippocampal destruction by blocking the accumulation of Aβ1–42 in the hippocampus.

Some studies have indicated that supplementation with carotenoids, omega-3 fatty acids, and vitamin E might improve working memory in old age (Salehi et al., 2018). Melatonin and its effects on sleep have also been explored in clinical trials for AD, with notable findings related to changes in sleep patterns, although direct cognitive or clinical improvements have yet to be firmly established, likely due to the short-term treatment periods (Meng and Wu, 2023).

Moving beyond AD, antioxidants in PD patients, especially omega 3/6, fatty acids, and vitamin supplements have been examined. These nutritional strategies comprise omega-3, omega-6, and traditional antioxidant vitamin formulations. Furthermore, new antioxidants like BN-82451 have been shown to enhance motility function and hinder neurodegeneration in mice models of HD (Salehi et al., 2018). In studies involving rodents, vitamin C and alpha-lipoic acid have displayed beneficial effects on locomotor symptoms and survival rates. Coenzyme Q10, which functions as a component of the mitochondrial membrane and a free radical scavenger, has demonstrated therapeutic potential in HD models. (Blyufer et al., 2021).

Riluzole is the only FDA-approved drug for treating ALS. Riluzole mitigates the generation of ROS by enhancing glutathione production, a pathway closely associated with mitochondria-driven apoptosis (Cho and Shukla, 2020). Recent placebo-randomized clinical trials have explored the use of Edaravone in ALS patients. Edaravone, recognized as the first free radical scavenger, offers potential benefits in modulating oxidative damage across various diseases, particularly neurodegenerative conditions (Sever et al., 2022).

In the context of ALS, novel compounds such as WN1316, an acylaminoimidazole derivative, have exhibited the ability to slow disease progression in advanced ALS mouse models. In HD, dietary flavonoid rutin was evaluated in a rat model, demonstrating potential therapeutic effects in this context (Khan et al., 2020).

### 5.5 Nanoparticles in neurodegenerative disease

Nanomedicine is a branch of medicine based on integrating nanotechnology which shows a huge potential for disease diagnosis and treatment. Nanomedicine offers a unique opportunity to administer drugs with selective targets in the brain via drug-loaded Nanoparticles(NPs) (Yusuf et al., 2023). The NPs can wrap around hydrophilic and hydrophobic drugs to increase their solubility and stability. More so, some of the inorganic or polymeric NPs developed can scavenge ROS and sequester iron thus offering a potential approach for mitigating iron overload-induced injuries and oxidative stress (Xu et al., 2022). Furthermore, nanomedicine has shown promise in modulating apoptotic pathways, both intrinsic and extrinsic and reducing the burden of Aβ-peptides, a critical initiator of AD. Exosomes, a type of NP, have been investigated as a reliable nanomedicine strategy because of their capability to traverse biological barriers and move to organs without blood supply (Chen et al., 2021). In a previous study, mesenchymal stem cell (MSC)-derived exosomes effectively restored the activity of genes linked to the adaptability of synapses and decreased the expression of Aβ in individuals with AD. Furthermore, the results indicated that mice subjected to this treatment demonstrated marked enhancements in cognitive abilities, alleviation of neuron and astrocyte damage, and improved brain glucose metabolism. Current treatments for AD involve using nanostructure-based delivery systems, which fall into four main categories: metallic/non-metallic NPs, organic NPs, lipid vesicles, and emulsion-based systems (Mir Najib Ullah et al., 2023). Gold NPs are optimal for crossing the BBB and have neuroprotective properties, making them effective in treating AD by inhibiting Aβ aggregation when combined with glutathione. Cerium-NPs also have neuroprotective potential and antioxidant effects, and when combined with triphenylphosphonium, they can prevent neuronal death and have an anti-Alzheimer effect (Mir Najib Ullah et al., 2023). Nanostructured lipid carriers are a promising delivery method for targeting drugs to the brain, with a disorganized inner lipid matrix of solid and liquid lipids. However, their physical instability and safety profile limit their use, which can be addressed by optimizing temperature, storage conditions, and pH levels (Mir Najib Ullah et al., 2023). Curcumin-loaded nanostructured lipid carriers are effective in treating oxidative stress conditions in AD by increasing curcumin bioavailability in the brain and reducing Aβ hallmarks (Ege, 2021). The use of indomethacin-containing lipid-core nanocapsules has been shown to reduce the harmful consequences caused by Aβ1-42 in patients with AD (Bernardi et al., 2012).

Nanophytomedicines for PD is a type of therapeutic agent that utilizes nanoparticles to deliver phytochemicals, such as those found in Mucuna pruriens, Ginkgo biloba, Turmeric, Green tea, and Ginseng, to the brain (Burns et al., 2023). These treatments aim to improve the efficacy and penetration of phytochemicals across the BBB, which is a major challenge in treating neurological diseases. *In vitro* studies often involve cell models to test the effectiveness of nanoplatforms and phytomedicines against PD on various molecular pathways implicated in the disease. For example, in one study, turmeric-initiated biocompatible gold nanoparticles increased cell viability in MPP + -treated PC-12 cells, a model for dopaminergic neurons (Burns et al., 2023). These nanoparticles exhibited antioxidative properties by scavenging free radicals and increasing antioxidant defense enzymes, thereby helping to balance or inhibit ROS generation at oxidative stress.

Vitro research has delved into the encapsulation of phytochemicals with nanoparticles to maximize their neuroprotective potential. A variety of NPs, such as gold, chitosan, and graphene oxide, have been utilized for this goal. Therapeutic agents, including curcumin, puerarin, ginkgolide B, and resveratrol, have exhibited the capacity to mitigate toxic effects on neuronal cells. Among these, curcumin is particularly effective in reducing α-synuclein aggregates, restoring cellular morphology, decreasing levels of ROS, and minimizing apoptosis (Burns et al., 2023).

Phytochemical encapsulation has been shown to enhance their bioavailability and neuroprotective effects, as compared to unencapsulated phytochemicals. Research indicates that encapsulation improves the blood-brain barrier permeability of phytomedicines, which is crucial for delivering therapeutic agents to the brain (Yang B. et al., 2020).

Although nanomedicine has shown promising results in enhancing the efficacy of medicines, reducing dosage requirements, and minimizing side effects in treating NDs, it has also been found to trigger inflammation, apoptosis, necrosis, autophagy, and oxidative stress, which may pose acute or long-term health risks (Fakhri et al., 2022). Therefore, it is essential to increase our understanding of the mechanism of toxicity and conduct further studies on NP-induced toxicity to ensure the safe use of nanomedicine in humans.

### 5.6 Genetics in neurodegenerative disease

Genetic therapy for NDs employs various techniques to modify or replace genes associated with these conditions, to address the underlying genetic causes of diseases such as AD, PD, and HD. This approach encompasses gene editing, replacement, or silencing strategies designed to either slow or potentially reverse disease progression. Recent works show the enrichment of rare variants in genes typically linked to early-onset, family degenerative diseases in populations affected by non-familial cases of the disease, which probably would imply a moderate effect on the risk of the disease (Dilliott et al., 2021). For instance, some rare variants associated with late-onset sporadic AD have been associated in patients with *APP, PSEN1*, and *PSEN2*; *SNCA, PARK2, LRRK2*, and *VPS35* have been associated with both early and late-onset sporadic *PD; SOD1, FUS* and *DNASC7* patients having both familial and sporadic ALS (Boros et al., 2022). One promising therapeutic avenue encompasses antisense oligonucleotides, the short nucleotide sequences, which can modulate gene expression, as well as splicing. Indeed, studies targeting *SOD1* and *C9orf72* mutations have demonstrated efficacy against preclinical models of ALS, and clinical studies using intrathecal administration of antisense oligonucleotides for these genes are ongoing (Sharma et al., 2017). Viral vectors are commonly utilized in gene therapy to facilitate the delivery of genetic material into cells, particularly in the treatment of NDs. These vectors have been engineered from viruses that possess the natural capability to infect and transfer their genomes into host cells (Rittiner et al., 2020). For instance, studies have shown that intracerebral administration of AAV2-NGF is well-tolerated and provides evidence of its therapeutic impact on cognitive decline in patients with AD-related dementia (Chen et al., 2020). In addition, a clinical trial has demonstrated the effectiveness of a lentiviral vector in delivering larger DNA payloads for PD. The study demonstrated that ProSavin, a gene therapy using lentiviral vectors to restore dopamine production, led to improvements in motor behavior and displayed safety in all patients with advanced PD (Chen et al., 2020). Non-viral vectors, despite their safety limitations, offer potential solutions. Exploring innovative vectors, such as nanoparticles and liposomes, is essential. Non-viral delivery vectors are categorized into lipid-based and polymeric vectors (Kanvinde et al., 2022). Another potential approach for transgene delivery involves gene silencing. RNA interference is a biological mechanism that involves the utilization of small interfering RNAs to decrease the synthesis of proteins by degrading their corresponding messenger RNAs. Multiple clinical trials have demonstrated that synthetic siRNAs can be administered to humans to specifically downregulate targeted genes or proteins, and are generally well-tolerated, as evidenced by studies such as NCT01559077 and NCT01437059 (Chen et al., 2020). While research on this field is ongoing and seems promising it remains mostly experimental for many neurodegenerative conditions.

### 5.7 Stem cell therapy in neurodegenerative disease

**FIGURE 5:**
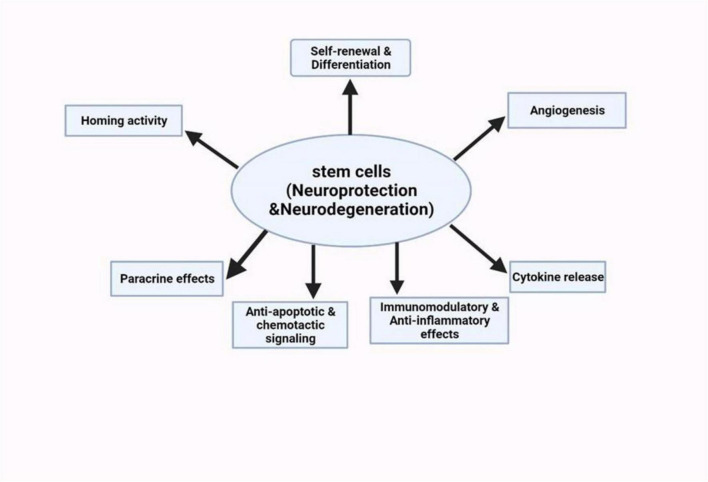
Schematic summary of the neuroprotective potentials of stem cell therapies on neurological disorders (Deming et al., 2018).

Stem cells are unique cell types with the ability to multiply extensively, regenerate, differentiate into various specialized cell types, and contribute to the regeneration of body tissues. It has a self-renewal capability and has also shown an ability to differentiate into diverse specialized cells (Biehl and Russell, 2009). These include embryonic stem cells, induced pluripotent stem cells, and neural stem cells. Stem cell therapy has emerged to be a potential area of research and treatment for NDs (Sivandzade and Cucullo, 2021). For a thorough grasp of how stem cell technology can be used to tackle NDs, we must know how different stem cell characteristics can interplay with the disease mechanisms (Figure 5). Currently, concerning AD and other similar neurodegenerative disorders, the main treatment strategies are based on adjusting neurotransmission activity, including acetylcholinesterase inhibition as an attempt to increase cholinergic efficacy (Hung and Fu, 2017). However, these treatments only provide temporary relief. Stem cell therapies can be used on the cholinergic system to produce an enriching environment. Studies have shown encouraging outcomes, such as intrathecal injection of human umbilical cord-derived MSCs into AD mouse models (Hernández and García, 2021). The use of this method was marked by a decrease in markers of glial activity, oxidative stress, and apoptosis in the mouse brain. The mice also showed cognitive enhancements in some areas which included learning and memory.

In another study, hematopoietic stem and progenitor cells were transplanted from mice with normal TREM2 function into mice with a defective TREM2 gene. This innovative approach resulted in the transplanted cells effectively reconstituting the blood system and integrating into the recipients’ brains, assuming the appearance and function of microglia (Deming et al., 2018). MSCs have shown encouraging outcomes in reducing symptoms in a mouse model of PD. This effect may be due to the secretion of neurotrophic factors, including brain-derived neurotrophic factor, glial cell-derived neurotrophic factor, and nerve growth factor, by mesenchymal stem cells (Palanisamy et al., 2023). These factors protect dopamine neurons from apoptosis and stimulate neurogenesis by releasing mitotic and proangiogenic substances. Dopamine progenitor cells derived from human pluripotent stem cells have yielded similar results in a primate PD model induced by 1-methyl-4-phenyl-1,2,3,6-tetrahydropyridine. These cells survived, matured, and functioned as midbrain dopamine neurons for an extended period without the formation of brain tumors. With higher risk management compared to MSCs-based therapies, clinical studies on human embryonic stem cell/induced pluripotent stem cell-derived cell products have produced good results and have been carried out in countries such as Australia, China, and Japan (Liu and Cheung, 2020). These acts showed that motor neurons had integrated and formed neural circuits in the host. However, moral and religious considerations about the use of fetal tissues are still important matters that must not be ignored as well as the possible risks such as the growth of the graft and the non-neuronal cells in the graft. One study that involved 11 ALS patients was conducted in Spain, and the results demonstrated growth in motor neuron counts following MSC transplantation, as well as an associated reduction in ubiquitin accumulation in the motor neurons. Currently, stem cell-based therapies are being considered as a potential solution for addressing the progressive loss of dopaminergic neurons, a hallmark of PD. Human pluripotent stem cells are viewed as a promising therapeutic approach, as they have the potential to differentiate into the necessary cell types for transplantation (Cha et al., 2023). While fetal cell transplantation has shown promise in preclinical and clinical studies, the use of human fetal cells has raised significant ethical and practical concerns. As a result, researchers are focusing on the use of human pluripotent stem cells as a less controversial source of cells for transplantation. However, there are still several challenges that need to be addressed, such as cell survival, integration into the host brain, immunological compatibility, and the risk of tumor formation (Cha et al., 2023). Researchers are actively working to overcome these challenges and refine the use of human pluripotent stem cells for cell replacement therapy.

Nanostructured lipid carriers are a developing method for effective drug delivery, consisting of an unorganized inner lipid structure with solid and liquid lipids. These carriers are used to deliver drugs to the brain for AD treatment, particularly in situations involving microglial activation. Nevertheless, their main limitations are their physical instability and safety concerns, which can be improved by optimizing temperature, storage conditions, and pH levels.

## 6 Conclusion

Over the past decade, extensive studies have focused on the role of neuroinflammation in neuronal degeneration. Accumulated evidence strongly supports the idea that neuroinflammation plays a pivotal role in both initiating and advancing the process of neurodegeneration and the subsequent loss of neurons in NDs. Furthermore, peripheral inflammation exacerbates neuroinflammatory pathways by activating various components, including glial cells, neurons, complement system, oxidative stress, and cytokines while increasing BBB permeability. Moreover, Suppression of neuroinflammation can alleviate the symptoms associated with NDs and limit the extent of neurodegeneration. Despite emerging therapeutic therapies that provide potentially effective treatment of NDs, a great deal has to be accomplished, and recent interest in preclinical studies and the first translations of some therapies into clinics has paved the way for further development in this direction.

## Author contributions

AA: Conceptualization, Project administration, Visualization, Writing – original draft. SL: Project administration, Resources, Writing – review and editing. FG: Supervision, Visualization, Writing – review and editing. GX: Conceptualization, Supervision, Writing – review and editing, Visualization.
